# Abdominal Manual Therapy Repairs Interstitial Cells of Cajal and Increases Colonic c-Kit Expression When Treating Bowel Dysfunction after Spinal Cord Injury

**DOI:** 10.1155/2017/1492327

**Published:** 2017-11-16

**Authors:** Yi Zhu, Yujie Yang, Jiabao Guo, Wenyi Zhang, Zhaojin Zhu, Bin Xie, Jun Yu, Jie Cheng

**Affiliations:** ^1^Rehabilitation Therapy Department, Hainan Provincial Nongken General Hospital, Haikou, Hainan 570100, China; ^2^The Second Clinical College, Nanjing University of Chinese Medicine, Nanjing, Jiangsu 210000, China; ^3^Rehabilitation Medicine Center, The First Affiliated Hospital of Nanjing Medical University, Nanjing, Jiangsu 210000, China; ^4^Rehabilitation Department, Yixing Jiuru Rehabilitation Hospital, Yixing, Jiangsu 214200, China; ^5^Department of Orthopedics & Traumatology, Jiangyin Orthopedics Hospital of Traditional Chinese Medicine, Jiangyin, Jiangsu 214400, China; ^6^Rehabilitation Department, Wuxi Hand Surgery Hospital, Wuxi, Jiangsu 214000, China

## Abstract

**Background:**

This study aimed to evaluate the therapeutic effects of abdominal manual therapy (AMT) on bowel dysfunction after spinal cord injury (SCI), investigating interstitial cells of Cajal (ICCs) and related c-kit expression.

**Methods:**

Model rats were divided as SCI and SCI with drug treatment (intragastric mosapride), low-intensity (SCI + LMT; 50 g, 50 times/min), and high-intensity AMT (SCI + HMT; 100 g, 150 times/min). After 14 days of treatment, weight, improved Basso-Beattie-Bresnahan (BBB) locomotor score, and intestinal movement were evaluated. Morphological structure of spinal cord and colon tissues were examined. Immunostaining, RT-PCR, and western blot were used to assess c-kit expression.

**Results:**

In SCI rats, AMT could not restore BBB, but it significantly increased weight, shortened time to defecation, increased feces amounts, and improved fecal pellet traits and colon histology. AMT improved the number, distribution, and ultrastructure of colonic ICCs, increasing colonic c-kit mRNA and protein levels. Compared with the SCI + Drug and SCI + LMT groups, the SCI + HMT group showed better therapeutic effect in improving intestinal transmission function and promoting c-kit expression.

**Conclusions:**

AMT is an effective therapy for recovery of intestinal transmission function. It could repair ICCs and increase c-kit expression in colon tissues after SCI, in a frequency-dependent and pressure-dependent manner.

## 1. Introduction

Spinal cord injury (SCI) can impair various sensations and movements, causing sphincter dysfunctions. More than one-third of SCI patients show constipation, prolonged defecation time, abdominal distention, and fecal incontinence, which further lead to decreased nutrition, limited movement, and increased mental stress [1]. 41% of SCI patients believe that bowel dysfunction seriously affects their quality of life and survival [2–4].

Currently, SCI management is merely supportive. Except for corticosteroids showing clinical benefits and spine surgery if warranted, active interventions are rather scarce [5]. Abdominal manual therapy (AMT) is widely used in clinic and safely increases colonic motility, shortens colonic transit time, and increases defecation frequency after SCI [6–11]. Nevertheless, optimal pressure intensity and frequency for AMT remain undetermined. In addition, the mechanisms underlying the therapeutic effects on bowel dysfunction after SCI remain unclear.

Gastrointestinal motility is coregulated by the central and enteric nervous systems. Upon SCI, the connection between the defecation nerve center (sacral spinal cord; S_2–4_) and central nerve center is lost, altering the normal function of the parasympathetic nerve system and damaging motor neurons in the enteric nervous system [12].

Interstitial cells of Cajal (ICCs) are pacemakers of gastrointestinal smooth muscle motility [13–15]. ICCs generate slow waves of rhythmic depolarization, which are transmitted to smooth muscle cells and spread to the distal end, thus promoting smooth muscle contraction. Meanwhile, ICCs mediate transduction of neural signals to smooth muscle cells [13, 16]. Therefore, ICCs are considered the “transit station” that transmits nerve impulses to smooth muscle cells [13, 17]. The density of ICCs is an important factor involved in the intestinal function [18]. Mature ICCs, as shown by high levels of ramification, indicate more efficient ICCs [19]. Tyrosine kinase receptor (c-kit) is a specific marker of ICCs and has stem cell factor (SCF) as a ligand; c-kit/SCF signaling is closely related to ICCs proliferation, differentiation, and phenotype maintenance [20, 21]. When c-kit is specifically blocked, the number of ICCs reversibly reduces [22]. Many diseases related to abnormal gastrointestinal motility alter ICCs ultrastructure, distribution, and/or number [17, 23–26]. Previous research pointed out that decreased activity of ICCs caused by SCI-induced enteric nervous dysfunction and may promote gastrointestinal dysmotility [12, 18]. Nevertheless, whether intestinal c-kit expression changes after SCI is unknown.

Based on the above findings, we hypothesized that managing bowel dysfunction after SCI by using AMT may involve restoring ICCs number and function, while increasing c-kit levels. This study aimed to assess the therapeutic effects of AMT on SCI-induced neurogenic bowel dysfunction, exploring ICCs changes in colon tissue.

## 2. Materials and Methods

### 2.1. Animals and Grouping

Ninety specific-pathogen-free (SPF) adult Sprague-Dawley (SD) rats (males, 45; females, 45; 200 ± 20 g) were obtained from Shanghai Sino-British Sippr/BK Lab Animal Co., Ltd. They had an improved Basso-Beattie-Bresnahan (Improved BBB) locomotor score [27] of 21 points. The rats were allowed one week of adaptation, housed under constant humidity 12/12 h light dark cycle at 25°C, with free access to food and water. The study was approved by the Ethics Committee of Nanjing University of Chinese Medicine.

Sixteen rats were randomly selected for sham operation. The remaining 74 animals underwent model establishment of severe SCI, and three died (two males, one female). Twenty-four hours postoperation, one female rat had an improved BBB score of 10 points and was excluded. Sixty-four of the 70 successful model rats were randomly assigned to the SCI, SCI + Drug, and low (SCI + LMT) and high (SCI + HMT) intensity manual therapy groups, respectively (*n* = 16).

Treatment began at 1 day postmodeling, and continued in the SCI + LMT/HMT and SCI + Drug groups for 14 days. The sham and SCI animals were fixed in the supine position for six minutes daily. Rat weights and Improved BBB scores were recorded for hind limb locomotor function evaluation at 15 days. Intestinal transmission function was measured at end of treatment.

At 16 days, the animals were sacrificed by cervical dislocation after deep anesthesia. The spinal cord at T_9–13_ was exposed. With T_11_ as the center, about 1.0 cm of fresh spinal cord tissue was removed. The rats were laparotomized. About 1 cm of proximal colon tissues (1-2 cm away from the cecum) was extracted for subsequent examinations.

### 2.2. Improved BBB Scoring

Hip, knee, and ankle joint movements, hind limb weight bearing capacity, walking ability, forelimb and hind limb coordination during walking, trunk stability, paw placement, and tail position were blindly assessed. The improved BBB scores were 0–21 points.

### 2.3. SCI Modeling

The weight-drop technique [27, 28] was used for SCI model establishment. 10% analytical pure chloral hydrate (Sinopharm Chemical Reagent Co., Ltd, China) was injected intraperitoneally (300 mg/kg) for anesthesia. Then, a cut on the median line was made from T_10_ to T_13_, with the animals in the prone position, exposing the spinous process and vertebral lamina. A micro mosquito clamp was used to remove the vertebral lamina from T_11_ to T_12_. Rats were fixed, followed by the drop of a 10-gram weight from 60 mm on the exposed spinal cord. After hemostasis, the layers were successively closed, with the surgical wound infiltrated with gentamycin.

In the sham group, the spinous process and vertebral lamina from T_10_ and T_13_ were exposed. Without injury treatment, the surgical wound was infiltrated with gentamycin and sutured directly.

### 2.4. Postoperative Care

For 16 postoperative days, 5000 U/kg of gentamycin was daily injected intraperitoneally. For the SCI animals, hind limbs were passively moved, with assisted urination performed every 12 h. The clinical Credé's maneuver [29] was used as reference to estimate urinary retention.

### 2.5. Abdominal Manual Therapy

In the SCI + HMT/LMT groups, rats were fixed in the supine position. A 5 mm diameter circular massage head with a smooth contact surface was used. Rats were rubbed in clockwise circles at bilateral areas 5 mm next to the umbilicus to simulate AMT with different intensities for 14 days. In the SCI + LMT group, pressure was 50 g at 50 times/min for 3 min/side. In the SCI + HMT group, pressure was 100 g at 150 times/min for 3 min/side.

### 2.6. Drug Treatment

In the SCI + Drug group, 0.02% mosapride (Sine, Pharmaceutical, Co., LTD, China) was administered by gavage at 1.5 mg/kg for 14 days.

### 2.7. Evaluation of Intestinal Transmission Function

Gastric instillation [30] was used to determine the time to first black fecal pellet. At 14 days, 8 rats/group were fasted for 24 h with only water allowed. Then, 2 mL of 100 g/L activated carbon suspension (Kelong Chemical Reagent Factory, China) was intragastrically administered. Time was recorded from gastric instillation to first black fecal pellet.

### 2.8. H&E Staining

Fresh spinal cord and proximal colon tissue specimens from five rats/group were fixed with 4% paraformaldehyde, followed by paraffin embedding. Conventional hematoxylin and eosin (H&E) staining was performed. Slides were mounted with neutral gum and analyzed by light microscopy.

### 2.9. Transmission Electron Microscopy

Proximal colon tissues (*n* = 5) were fixed in 5% glutaraldehyde for 2 h at 4°C, postfixed in 1% osmic acid (2 h at 4°C), and stained with 2% uranyl acetate. The slides were dehydrated in graded ethanol at 4°C. After toluidine blue staining, the specimens were sliced into semithin sections (70–80 nm) on an ultramicrotome (Leica RM 2145, Germany). Electron microscopy at 4000–25,000x magnification (JEM-1010 transmission electron microscope, JEOL Co., Japan) was performed to observe the changes in colonic ICC ultrastructure.

### 2.10. Immunohistochemistry

Proximal colon tissues (*n* = 5) were paraffin embedded and sliced (5 *μ*m). Staining was performed with a ready-to-use rapid immunohistochemistry MaxVision™ kit (Fuzhou Maixin Biotech. Co., Ltd., China), according to the manufacturer's instructions. The c-kit primary antibody was purchased from Santa Cruz Biotechnology (USA) and used at 1 : 100 dilution. Slides were imaged by light microscopy. Ten high-power fields were randomly selected per sample for analysis. The JD801 image analysis system (Nanjing Jieda Company) was used to determine average optical density (AOD) values of positive staining areas.

### 2.11. Real-Time PCR

Proximal colon tissues (*n* = 5) were frozen in liquid nitrogen. RNA was extracted with RNA Isolater Total RNA Extraction Reagent (Vazyme Biotech Co., Ltd., China) and reverse-transcribed with HiScript™ Q RT SuperMix for qPCR (Vazyme Biotech Co., Ltd.), according to the manufacturers' instructions. With GAPDH as internal control, quantitative PCR was performed using AceQ™qPCR SYBR® Green Master Mix (Vazyme Biotech) on ABI StepOne™ Plus (Applied Biosystems, USA). Primers synthesized by Vazyme Biotech were as follows: c-kit, sense 5′-CCTCGCCTCCAAGAACTGTATT-3′ and antisense 5′-GCCGTGCATTTCCTTTTACC-3′; GAPDH, sense 5′-GAGTCCACTGGCGTCTTCA-3′ and antisense 5′-GGGGTGCTAAGCAGTTGGT-3′. PCR was performed for 40 cycles at 95°C (5 min), 95°C (10 s), and 60°C (30 s). Data were analyzed by the 2^−ΔCT^ method.

### 2.12. Western Blot

Proximal colon tissues (*n* = 4) were lysed with the RIPA lysis buffer (Vazyme Biotech). Protein amounts were quantified by the BCA method. Total proteins (170 *μ*g) were separated by SDS-PAGE followed by being transferred onto PVDF membranes. After blocking, the membranes were incubated with primary antibodies against c-kit (Santa Cruz; 1 : 200) and tubulin (Vazyme Biotech; 1 : 5000), respectively. Then, secondary antibodies were added for 1 h at room temperature (1 : 5000). After chemiluminescent reaction, the membranes were analyzed on a ChemiScope chemiluminescence imaging system (ClinX Science Instruments Co., Ltd, China). Chemical analysis (Gel Analysis V2.02) was used to assess grey values of the immunoreactive bands. c-Kit expression was normalized to tubulin amounts.

### 2.13. Statistical Methods

SPSS 16.0 (SPSS, USA) was used for statistical analysis. Data were presented as mean ± standard deviation (SD). Normally distributed data were analyzed by one-way analysis of variance (ANOVA), with LSD (assumption of homogeneity of variance) or Dunnett's T3 (nonassumption of homogeneity of variance) for pairwise comparisons. Paired* t*-test was used for comparing body weights. *p* < 0.05 was considered statistically significant.

## 3. Results

### 3.1. AMT Improves the Overall Condition of Animals after SCI

**Figure 1:**
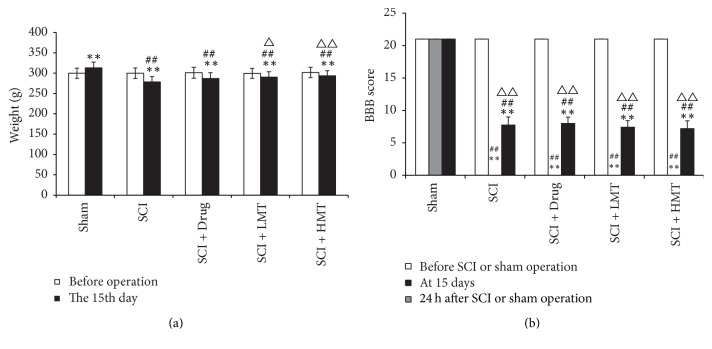
*Overall conditions of animals in different groups*. (a) Animal weight was measured before and 15 days after spinal cord injury (SCI) or sham operation in the sham, SCI, and SCI with mosapride treatment (SCI + Drug), low-intensity manual therapy (SCI + LMT), and high-intensity manual therapy (SCI + HMT) groups (*n* = 16/group). ^*∗∗*^*p* < 0.01, compared with the preoperative values; ^##^*p* < 0.01, compared with the sham group; ^△^*p* < 0.05 or ^△△^*p* < 0.01, compared with the SCI group. (b) The improved BBB score was assessed before and 24 hours or 15 days after SCI or sham operation in the sham, SCI, SCI + Drug, SCI + LMT, and SCI + HMT groups (*n* = 16/group). ^*∗∗*^*p* < 0.01, compared with the sham group; ^##^*p* < 0.01, compared with the preoperative values; ^△△^*p* < 0.01, compared with the 24 h values.

Before SCI or sham operation, animal weight in the five groups had no significant differences (Figure 1(a)). At 15 days, body weight in sham animals was higher than the preoperative values (*p* < 0.01). In the SCI, SCI + Drug, SCI + LMT, and SCI + HMT groups, animal weight was decreased (all *p* < 0.01). Body weight in the SCI group was significantly reduced compared with the sham group (*p* < 0.01). Interestingly, the SCI + LMT and SCI + HMT groups showed significantly higher body weight compared with the SCI group (*p* < 0.05 and *p* < 0.01, resp.).

Sham animals showed glowing hair, normal fitness, and locomotor activity, with usual food and water intake, and independent defecation and urination. In contrast, SCI rats had pale, messy and loose hair and were restless and occasionally with strong aggressiveness, and reduced locomotor activity. Hind limbs showed varying degrees of muscle atrophy. Food intake and defecation amounts were reduced, with different degrees of urinary retention. At 15 days, sham animals had a BBB score of 21 points, while the scores in the remaining four groups were 6–9 points (Figure 1(b)).

### 3.2. AMT Ameliorates Intestinal Transmission Function after SCI

**Figure 2:**
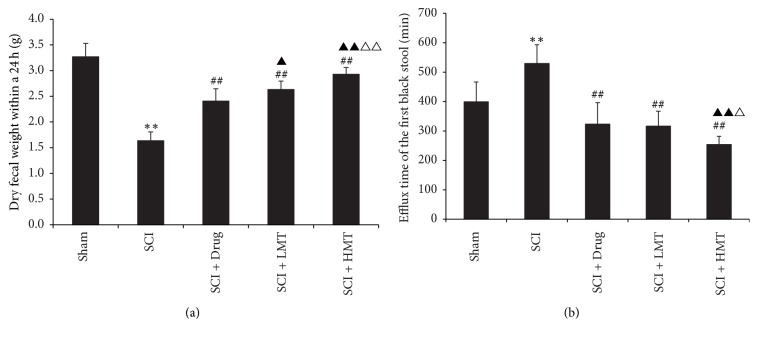
*Intestinal transmission functions in different groups*. After therapy, the 24 h weight of dry feces (a) and time to first black fecal pellet after activated carbon administration (b) were measured in the sham, SCI, SCI + Drug, SCI + LMT, and SCI + HMT groups (*n* = 8). ^*∗∗*^*p* < 0.01, compared with the sham group; ^##^*p* < 0.01, compared with the SCI group; ^▲^*p* < 0.05 or ^▲▲^*p* < 0.01, compared with the SCI + Drug group; ^△^*p* < 0.05 or ^△△^*p* < 0.01, compared with the SCI + LMT group.

At 16 days, sham rats showed easy defecation with fecal pellets appearing as soft and moist elongated grains. Rats in the SCI group had difficult defecation; fecal pellets were smaller, hard, and dry grains. Feces amounts were significantly reduced in the SCI group compared with sham animals (*p* < 0.01) (Figure 2(a)). The SCI + Drug group had smooth bowel movement, but most fecal pellets showed a granular shape. Occasionally, there were irregular fecal pellets discharged. Feces amounts were larger than that of the SCI group (*p* < 0.01). In the SCI + LMT and SCI + HMT groups, bowel movement was smooth. Fecal pellets were slightly smaller than those of sham rats and elongated. Single pellet or 2-3 fecal pellets were defecated, with relatively hard texture. Feces amounts were larger than those of the SCI and SCI + Drug groups (all *p* < 0.05). Feces amounts in the SCI + HMT group were larger than that of the SCI + LMT group (*p* < 0.01) (Figure 2(a)).

There were significant overall differences in times to first black fecal pellet in the five groups (*p* < 0.01). Compared with the SCI group, the SCI + Drug, SCI + LMT, and SCI + HMT groups showed shortened times (all *p* < 0.01). Compared with the SCI + Drug and SCI + LMT groups, time to first black fecal pellet in the SCI + HMT group was significantly shorter (*p* < 0.01 and *p* < 0.05, resp.) (Figure 2(b)).

### 3.3. AMT Restores SCI-Induced Pathological Changes in Colon but Not in Spinal Cord Tissues

**Figure 3:**
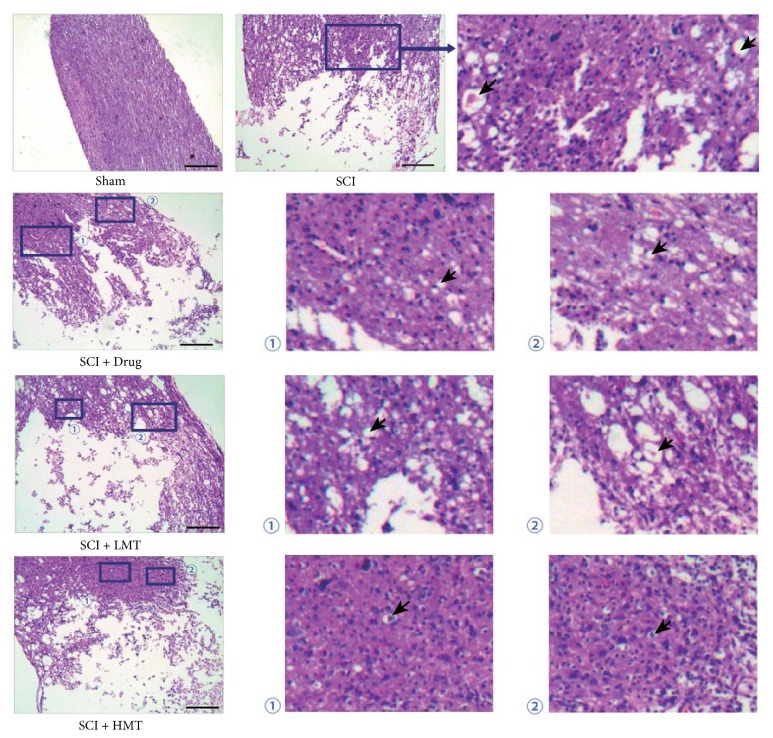
*Morphology (H&E staining) of spinal cord tissues in different groups*. After therapy, the morphology of spinal cord tissues was assessed by H&E staining in the sham, SCI, SCI + Drug, SCI + LMT, and SCI + HMT groups (*n* = 5). Arrow indicates edema. Scale bar, 1.35 mm.

As shown in Figure 3, spinal cord tissues in the sham group had intact structure. Spinal cord tissues of rats in the remaining four groups had overt damage, with disordered structure and complete nerve cell necrosis, incomplete surrounding structure, and significant edema and degeneration of some cells.

**Figure 4:**
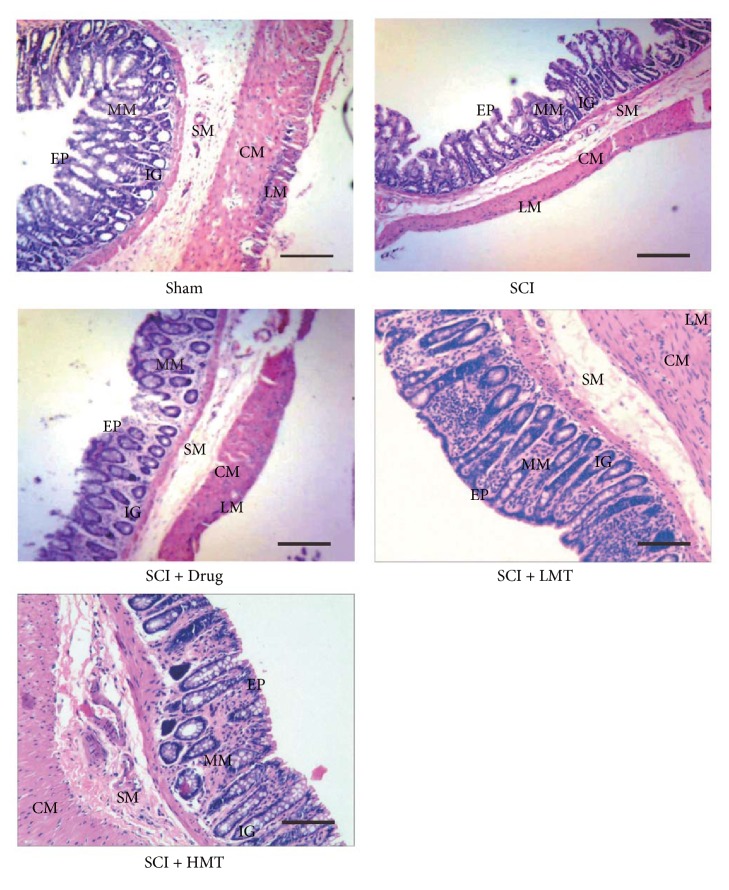
*Morphology (H&E staining) of colonic tissues in different groups*. After therapy, the morphology of colon tissues was assessed by H&E staining in the sham, SCI, SCI + Drug, SCI + LMT, and SCI + HMT groups (*n* = 5). LM, longitudinal muscle; CM, circular muscle; SM, submucosa; IG, inherent glands; MM, mucous membrane; EP, epithelium. Scale bar, 1.35 mm.

Compared with the sham group, the SCI group showed overt intestinal wall atrophy and mucosal erosion, together with inflammatory cell infiltration, reduction of inherent glands, significant muscle atrophy, and mild interstitial edema. In the SCI + Drug group, overt intestinal wall atrophy, mucosal erosion, inflammatory cell infiltration, inherent gland reduction, and muscle atrophy were observed. Colon morphology in the SCI + Drug group was closer to that of the SCI group. In the SCI + LMT and SCI + HMT groups, slight atrophy of the muscle layer and inherent glands were observed. Colon morphology in these two groups was closer to that of the sham animals (Figure 4).

### 3.4. AMT Restores the Morphology of Colonic ICCs after SCI

**Figure 5:**
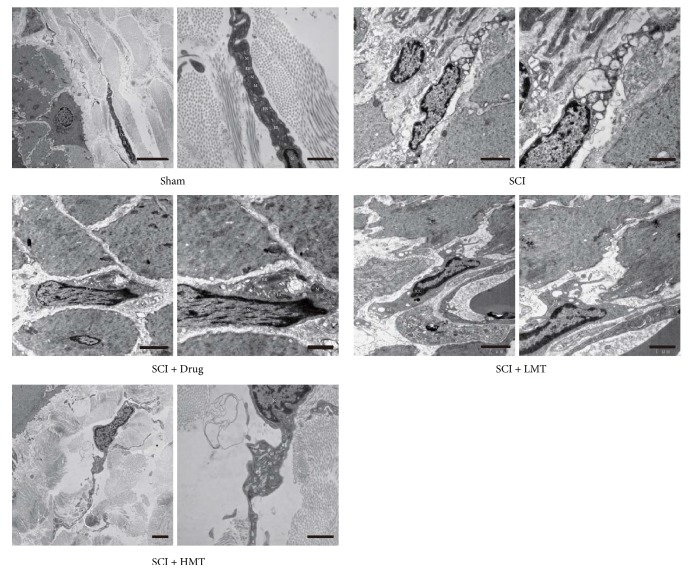
*Ultrastructure of colon ICCs in different groups*. After therapy, the ultrastructure of colon tissues was assessed by transmission electron microscopy (TEM) in the sham, SCI, SCI + Drug, SCI + LMT, and SCI + HMT groups (*n* = 5). ICC, interstitial cell of Cajal; N, nucleus; M, mitochondria; ER, endoplasmic reticulum. Scale bar: 5 *μ*m (left) and 1 *μ*m (right) for the sham group; 2 *μ*m (left) and 1 *μ*m (right) for the SCI, SCI + Drug, SCI + LMT, and SCI + HMT groups.

As assessed by electron microscopy (Figure 5), colonic ICCs in the sham group were spindle-shaped with large and obvious nuclei. The chromatin was dispersed throughout the nucleus. There were abundant mitochondria, endoplasmic reticulum, and Golgi apparatus in the cytoplasm.

Colonic ICCs in the SCI group had irregular cell morphology with vacuoles in the cytoplasm. The ultrastructure of some cells was undefined. The number of organelles was significantly decreased, and structural abnormalities appeared. Mitochondria were decreased in number, with swelling, dissolution, and even rupture and vacuole formation. Expansion and degranulation of the endoplasmic reticulum were observed, and lipid droplets appeared.

Colonic ICCs in the SCI + Drug group showed improved morphology compared with those of the SCI group. There were vacuoles in the cytoplasm with relatively complete organelle structure. Mitochondria were swollen. Endoplasmic reticulum was expanded.

Colonic ICCs in the SCI + LMT and SCI + HMT groups were spindle-shaped and similar to those of sham animals. Vacuoles were found in the cytoplasm with clear nucleus. More organelles were found compared with the SCI group and morphological structure was relatively complete. Mitochondria were slightly swollen, with expanded endoplasmic reticulum. Overall, the ultrastructural properties of colonic ICCs in the SCI + Drug, SCI + LMT, and SCI + HMT groups were similar.

### 3.5. AMT Increases the Level of Colonic c-Kit Expression after SCI

**Figure 6:**
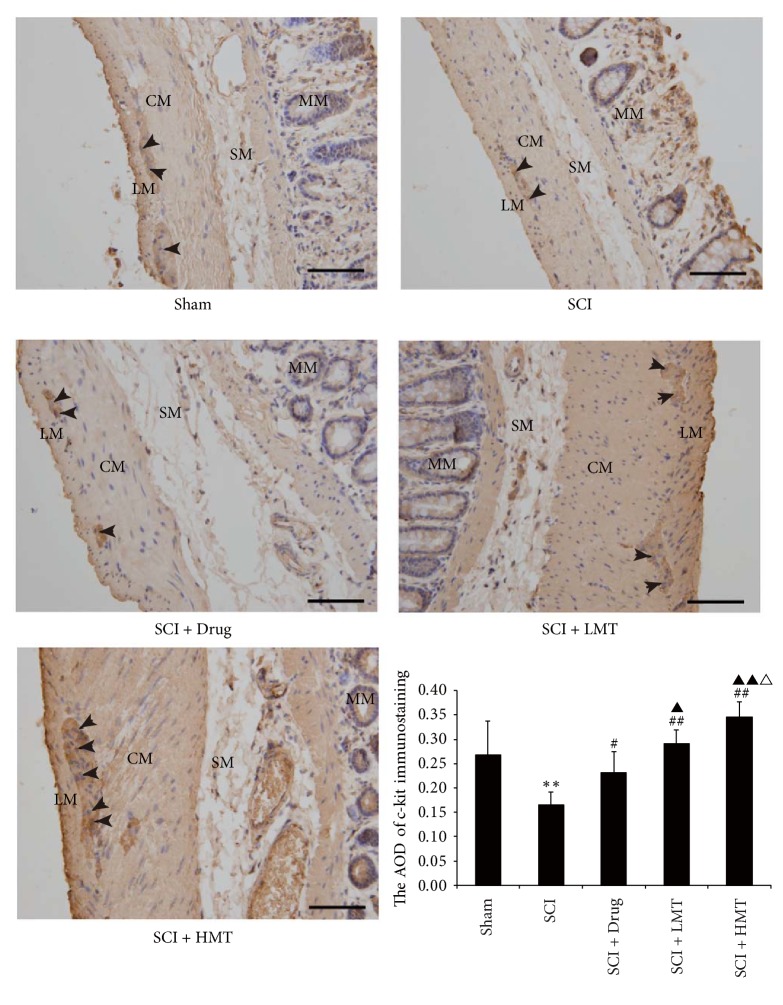
*Immunochemistry of colonic c-kit expression in different groups*. After therapy, the expression of c-kit was assessed by immunohistochemistry in the sham, SCI, SCI + Drug, SCI + LMT, and SCI + HMT groups (*n* = 5, 10 fields/slide). LM, longitudinal muscle; CM, circular muscle; SM, submucosa; MM, mucous membrane. Arrow indicates c-kit positive cells which were considered as ICCs. AOD, average optical density. Scale bar, 135 *μ*m. ^*∗∗*^*p* < 0.01, the SCI group compared with the sham group; ^#^*p* < 0.05 or ^##^*p* < 0.01, compared with the SCI group; ^▲^*p* < 0.05 or ^▲▲^*p* < 0.01, compared with the SCI + Drug group; ^△^*p* < 0.05, compared with the SCI + LMT group.

As shown by immunohistochemistry (Figure 6), in the sham group, c-kit positive ICCs were densely distributed. In the SCI group, the c-kit expression was significantly reduced. c-Kit expression in the SCI + Drug group was increased significantly compared with the SCI group. The SCI + LMT and SCI + HMT groups also showed significantly higher c-kit expression compared with the SCI group.

AOD values for c-kit immunohistochemistry among the five groups were significantly different (*p* < 0.01) (Figure 6). Compared with sham animals, AOD values of the SCI group were lower (*p* < 0.01). Compared with the SCI group, AOD values in the SCI + Drug, SCI + LMT, and SCI + HMT groups were significantly increased (*p* < 0.05, *p* < 0.01 and *p* < 0.01, resp.). Compared with the SCI + Drug group, AOD values in the SCI + HMT/LMT groups were significantly increased (*p* < 0.05 and *p* < 0.01, resp.). Finally, AOD values in the SCI + HMT group were significantly higher than those of the SCI + LMT group (*p* < 0.05) (Figure 6).

**Figure 7:**
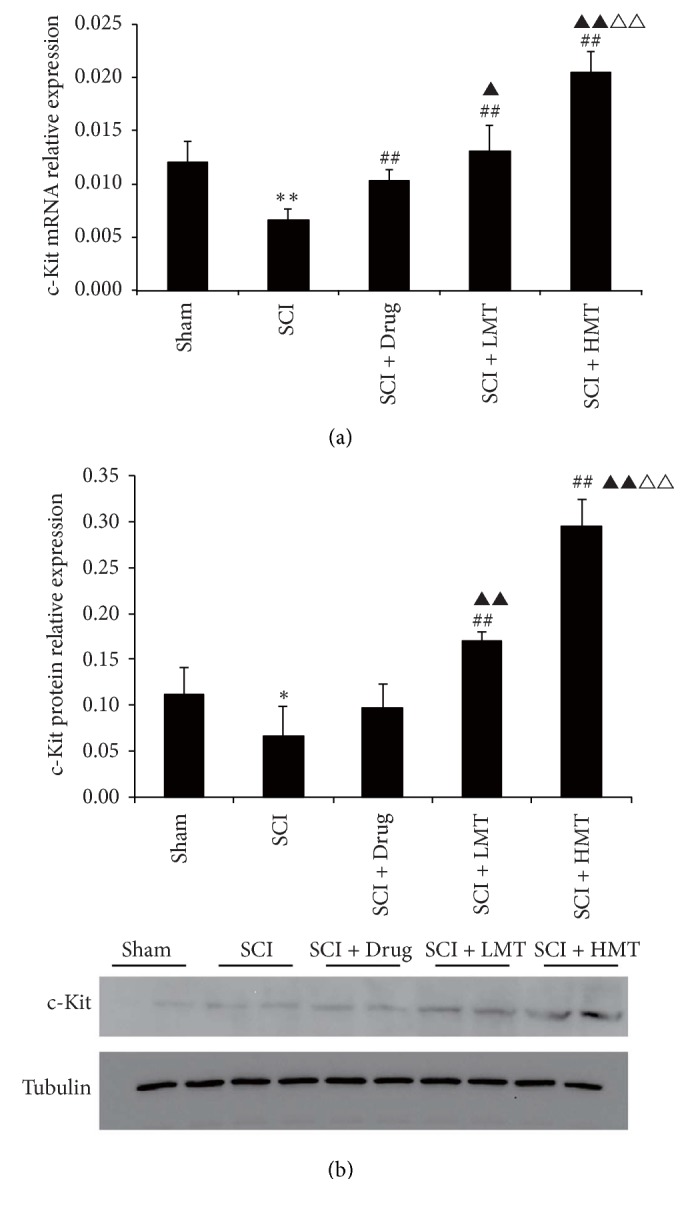
*Gene and protein expression levels of c-kit in different groups*. After therapy, the gene expression levels of c-kit were evaluated by RT-PCR (a), normalized with the internal control GAPDH (*n* = 5). Western blot was used for c-kit protein level assessment, with tubulin as a loading control (*n* = 4). The sham, SCI, SCI + Drug, SCI + LMT, and SCI + HMT groups were analyzed. Representative western blot images are displayed in the bottom panel. ^*∗∗*^*p* < 0.01, the SCI group compared with the sham group; ^##^*p* < 0.01, compared with the SCI group; ^▲^*p* < 0.05 or ^▲▲^*p* < 0.01, compared with the SCI + Drug group; ^△△^*p* < 0.01, compared with the SCI + LMT group. ^*∗*^*p* < 0.05.

Colonic c-kit mRNA levels in the five groups showed significant differences (*p* < 0.01). Compared with sham animals, relative gene expression of c-kit in the SCI group was decreased significantly (*p* < 0.01). c-Kit mRNA levels in the SCI + Drug and SCI + HMT/LMT groups were increased compared with the SCI group (both *p* < 0.01). Compared with the SCI + Drug group, c-kit mRNA amounts in the SCI + HMT/LMT groups were higher (*p* < 0.05 and *p* < 0.01, resp.). Meanwhile, c-kit mRNA levels in the SCI + HMT group were significantly higher than in the SCI + LMT group (*p* < 0.01) (Figure 7(a)).

Colonic c-kit protein amounts in the five groups also showed significant differences (*p* < 0.01) (Figure 7(b)). Compared with sham animals, c-kit protein amounts in the SCI group were decreased significantly (*p* < 0.05). Notably, c-kit protein levels in the SCI + HMT/LMT groups were increased significantly compared with the SCI group (both *p* < 0.01), while the SCI + Drug and SCI groups showed similar values. Meanwhile, c-kit protein expression in the SCI + HMT group was significantly higher than that of the SCI + LMT group (*p* < 0.01) (Figure 7(b)).

## 4. Discussion

In this study, the T_11-12_ segment was injured for model establishment, and the injury level was above the conus medullaris (upper motor neuron injury). The bowel dysfunction in the SCI models generated here should mainly be manifested as extended colonic transit time, delayed transmission, and difficult bowel movement [4, 31]. In line with the theoretical expectations, the SCI rats showed reduced weight, dry stool, difficult bowel movement, prolonged defecation, reduced feces amounts, and intestinal transit disorder. Mosapride is a prokinetic therapeutic for dyspeptic symptoms of gastrointestinal disorders [32]. Although mosapride treatment shortened defecation time in rats with SCI to some extent and increased feces amounts, its improvement of fecal pellet traits was not significant. Interestingly, AMT with different intensities showed more advantages than mosapride treatment in shortening defecation time, increasing feces amounts, and improving stool traits, and is worthy of clinical attention.

Morphologically, SCI caused a significant damage in rat spinal cord continuity, with complete local nerve cell necrosis, incomplete surrounding structure, and cell edema and degeneration, corroborating previous observations [33, 34]. Notably, neither AMT nor mosapride could restore spinal cord tissues. As for morphologic features of rat colon tissues, after SCI, the intestinal cavity was expanded with intestinal wall structure also undergoing corresponding pathology changes, which may result from stool accumulation. Nevertheless, AMT and the gastrointestinal excitomotor mosapride restored colon tissue morphology, with the former being more effective. These findings indicated that such treatments, especially AMT, could improve intestinal transit function and reduce intestinal fecal accumulation.

The density of ICCs is an important factor involved in the intestinal function [18]. Mature ICCs, as shown by high levels of ramification, indicate more efficient ICCs [19]. In addition, neurotransmitters from enteric nerves activates ion channels in ICCs [12]. In the present study, ultrastructural assessment revealed significantly less colonic ICCs in SCI rats compared with sham animals. Meanwhile, abnormal ultrastructure was observed in SCI rats. The c-kit protein and its ligand SCF constitute the c-kit signaling system, which is closely related to proliferation, differentiation, and maintenance of the ICC phenotype [20, 21]. As shown above, colonic c-kit protein expression in rats with bowel dysfunction after SCI was reduced compared with that of sham animals. Similar data were obtained at the gene level. The changes in ICC morphology could be due to a lesser excitation from neurotransmitters [12]. These findings suggest that while reduced number and damaged ultrastructure of colonic ICCs and low expression of colonic c-kit may not initially cause neurogenic intestinal dysfunction, they may promote bowel dysfunction worsening after SCI. AMT treatment could alleviate the pathological changes of ICCs and increase c-kit expression in colon tissues of SCI rats. Although mosapride treatment also helped repair colonic ICCs, its effects on promoting c-kit expression were not as powerful as AMT, especially when compared to SCI + HMT.

In this study, we compared the therapeutic effects of different AMT intensities, and SCI + HMT was generally more potent than SCI + LMT in alleviating the various SCI-induced symptoms. For efficacy to be achieved with minimal discomfort to animals, therapeutic pressure and frequency were set in preliminary experiments. SCI + HMT and SCI + LMT groups were submitted to 100 g at 150 times/min and 50 g at 50 times/min, respectively. As shown above, AMT effects on bowel dysfunction after SCI were frequency- and pressure-dependent. As proposed by our results, the possible mechanisms underlying the therapeutic effects of AMT may be related to the changes of ICCs, including their amounts and functions, as indicated by c-kit expression [22, 23]. Neurogenic bowel dysfunction after spinal cord injury leads to the accumulation of feces and luminal dilatation, which in turn aggravates the damage to intestinal morphology and functions [30]. AMT to the abdomen could provide an appropriate mechanical stimulus to promote the movement of the intestinal tract, increasing defecation and thus preventing further damage to the cells regulating gastrointestinal motility, such as ICCs [35–37]. On the other hand, the enhanced proliferation and functions of ICCs may also be a direct result of the mechanical stimulus during AMT [35–37]. Taken together, apart from mechanical stimulus-induced intestinal movement, the protection and/or activation of ICCs by AMT may help recover the gastrointestinal autonomic rhythmicity, therefore further improving bowel dysfunction. Nevertheless, our study was mainly designed to examine the impact of AMT on ICCs; more other exact comprehensive mechanical and molecular mechanisms involved in this process are necessary to address in additional studies in the future. Moreover, AMT should be further standardized and evaluated in clinical trials.

In conclusion, AMT repaired ICCs and increased c-kit expression in colon tissues after SCI, in a frequency- and pressure-dependent manner. This may be an important mechanism by which AMT promotes recovery of intestinal transmission function in rats after SCI.
